# Iodine-125 brachytherapy in inoperable duodenal papilla carcinoma: a case report series

**DOI:** 10.3389/fonc.2024.1394178

**Published:** 2024-07-10

**Authors:** Yue Zhang, Shangbin Xu, Jing Xu, Shen Wu, Wenyi Yao, Shengying Lu, Guangtao Zhang, Tingsong Chen

**Affiliations:** ^1^ Department of Interventional Oncology, Seventh People’s Hospital of Shanghai University of Traditional Chinese Medicine, Shanghai, China; ^2^ Department of Oncology, Longhua Hospital, Shanghai University of Traditional Chinese Medicine, Shanghai, China

**Keywords:** duodenal papilla carcinoma, 125 I seed implantation, brachytherapy, interventional therapy, alternative therapy

## Abstract

**Background:**

Duodenal papilla carcinoma (DPC) is a rare malignancy often diagnosed at an advanced stage. When surgery is not feasible in localized disease due to advanced age or comorbidities, there remains no consensus on optimal management for these patients.

**Case summary:**

This case series details the therapeutic outcomes of ^125^I seed implantation in two elderly patients with DPC. A notable tumor reduction was achieved within two months after implantation. Furthermore, both patients demonstrated radiological tumor response and survived for over six months following the initial ^125^I seed treatment, marking the first reported instance of ^125^I seed implantation to effectively control DPC.

**Conclusion:**

The anti-tumor activity of ^125^I seed implantation in the reported two cases of DPC underscores its potential as a viable treatment option for inoperable localized DPC.

## Introduction

Duodenal papilla carcinoma (DPC) is a rare malignancy, accounting for only 1% of all malignancies originating from epithelial cells (1, 2). Previous research has consistently underscored the significant potential of surgical intervention in DPC cases. Pancreaticoduodenectomy stands as the primary therapeutic approach for this condition (3, 4). However, surgical resection is usually contraindicated in localized DPC patients due to advanced age or concurrent comorbidities. Alternative management approaches, such has external beam radiotherapy and/or systemic chemotherapy and/or palliative care are recommended. Individualized locoregional strategies are optional to frail patients who are intolerant to active anti-tumor treatments.

Iodine-125 (^125^I) seed implantation has emerged as a breakthrough in locoregional treatments (5, 6). This brachytherapy is a novel internal radiation therapy that differs from the conventional external beam radiation therapy (EBRT) by providing a continuous, low-energy radiation source, in which X-rays and gamma rays are continuously emitted with high conformability (7). Despite its application across various cancers, the efficacy of ^125^I seed brachytherapy on DPC remains unexplored. Herein, we reported the successful application of ^125^I seed implantation to two elderly DPC patients, showing a remarkable tumor response in both cases.

## Methods

### ^125^I seed characteristics and dosimetry estimation

The ^125^I seeds, sourced from Shanghai Xinke Pharmaceutical Co., Ltd (Shanghai, China), are encapsulated within a durable titanium casing that possess a half-life of 60.1 days. Each seed exhibits a radioactivity level of 0.786 mCi, primarily emitting 27.4 and 31.4 keV X-rays along with 35.5 keV gamma rays. The optimal number of seeds for implantation was determined according to the American Association of Physicists in Medicine TG43U1 brachytherapy formalism (a calculation protocol of low energy brachytherapy interstitial sources) and the individualized condition of malignant obstructive jaundice, using the following formula: n=the maximum length of the lesion (mm)/4.5 + 4 (8, 9). The ^125^I seeds are removed after 3-9 months of implantation.

### Percutaneous transhepatic ^125^I seed implantation

A set of biochemical and hematological examinations were carried out before and after the procedure. After local anesthesia with lidocaine, a percutaneous transhepatic cholangiodrainage (PTCD) channel was established. A 4F catheter was used to make an ^125^I seed strand and delivered to the target position under fluoroscopic supervision. Written informed consent of each patient was obtained before surgery.

## Case presentation

### Case 1

**Figure 1:**
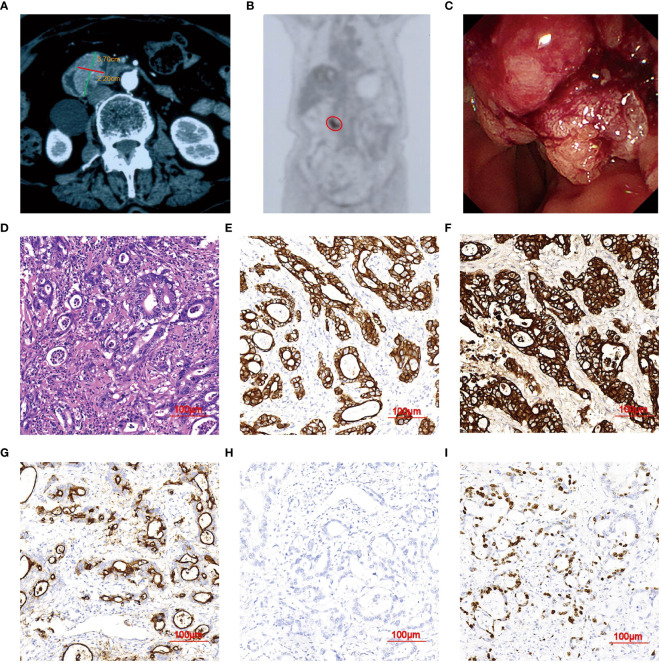
Imaging Features of DPC in case 1. **(A)** Abdominal CT scan showing a nodule (3.7×2.2 cm). **(B)** PET/CT images demonstrating a single focus in the upper abdomen (red circle). **(C)** ERCP revealing an elevated lesion measuring 3.7 mm in diameter at the duodenal papilla of Vater. **(D)** H&E staining indicating poorly-differentiated adenocarcinoma of DPC (magnification, ×20). **(E–H)** IHC showing tumors positive for CK7, CK19, and MUC1, and negative for MUC2. **(I)** Apoptosis index. Ki-67 positive rate was about 50%.

An 80-year-old female was admitted to a local hospital, presenting with a one-week history of fever in September 2022. Contrast-enhanced abdominal computed tomography (CT) revealed a heterogeneous, hypo-dense lesion in the liver, measuring approximately 6 cm in diameter, indicative of an abscess. The fever resolved following percutaneous abscess drainage and antibiotic therapy. Additionally, a solid mass measuring 3.7×2.2×1.7cm was detected at the ampulla of Vater (**Figure 1A**). ^18^F-FDG PET/CT scan exhibited an intense FDG uptake in the duodenal mass, suggestive of malignancy (**Figure 1B**). Subsequent endoscopic retrograde cholangiopancreatography (ERCP) revealed a large tumor at the papilla of Vater, from which multiple biopsies were taken (**Figure 1C**). Histological examination showed abundant malignant epithelial cells characterized by cuboidal cells with rounded nuclei arranged in a monolayer, accompanied by infiltrative interstitial inflammatory cells (**Figure 1D**). Immunohistochemistry (IHC) staining was positive for CK7, CK19, MUC1, and Ki67 (40%), and negative for MUC2 (**Figures 1E–I**). A pathological diagnosis of poorly differentiated adenocarcinoma of DPC, in the pancreaticobiliary subtype according to IHC results.

**Figure 2:**
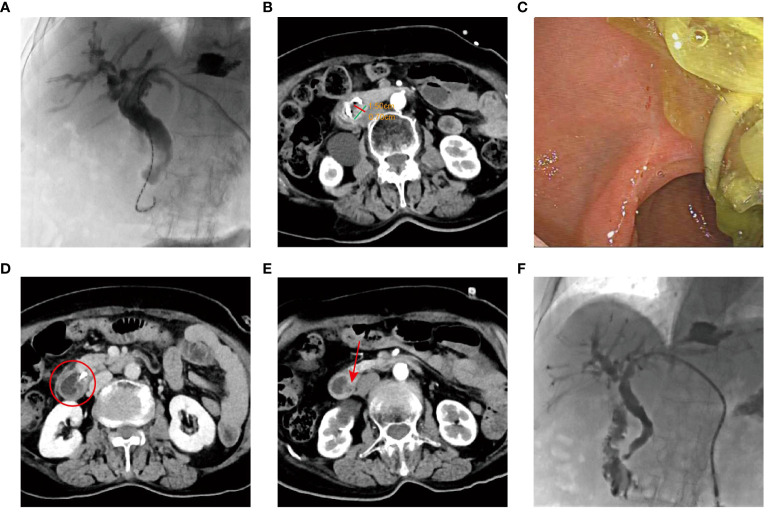
The examination results of Case 1 after ^125^I implantation. **(A)** Placement of ^125^I seed strands on the tumor surface. **(B)** Three months after ^125^I seed implantation, enhanced CT indicated the treatment response was PR. **(C)** Three months after brachytherapy, duodenoscope revealing disappearance of the lesions, with no duodenal obstruction. **(D)** Eight months after ^125^I seed implantation, tumor disappeared (the red circle). **(E)** Ten months after ^125^I seed implantation, no recurrence (the arrow indicates the duodenal papilla). **(F)** The images of duodenography and cholangiography.

The patient was referred to our hospital in October 2022. She denied a history of hypertension, diabetes, hepatitis, psychiatric and inherited disease. However, the patient had a history of gallstones and cholecystectomy was performed in a local hospital in July 2021. A physical examination revealed normal vital signs. Biochemical testing showed plasma carbohydrate antigen 19-9 (CA19-9) level of 20.49 µ/mL (normal range < 27 µ/mL), carcinoembryonic antigen (CEA) of 1.95 ng/mL (normal range < 5 ng/mL), and total bilirubin (TBIL) of 38.4 μmol/L (normal range ≤ 23 μmol/L). The Karnofsky Performance Scale (KPS) was 40. The radiological staging was determined at TxN0M0 by the current 8^th^ edition of the American Joint Committee on Cancer (AJCC) staging system for Ampulla of Vater cancers. Given the advanced age and poor performance status, the patient was not suitable for surgery or chemotherapy. After obtaining the patient’s informed consent, brachytherapy was scheduled. Subsequently, the patient underwent percutaneous transhepatic cholangiodrainage (PTCD) on October 21, 2022, followed by ^125^I seed implantation (**Figure 2A**). According to the revised Response Evaluation Criteria in Solid Tumors (RECIST) guideline (version 1.1) (10), a partial response (PR) was observed by January 2023, with a 59.5% reduction in the maximum diameter (decreased from 3.7 cm to 1.5 cm) (**Figure 2B**). Biochemical testing showed that TBIL decreased below upper normal limits to 13.8 μmol/L, and KPS increased to 60. By June 2023, a complete response (CR) was achieved, with no tumor identified via duodenoscopy and CT imaging (**Figures 2C, D**). Biochemical testing showed that plasma TBIL was 23.9 μmol/L. KPS further increased to 80. The patient was barely able to carry out normal activities, but occasionally had distension pain in the upper abdomen. The latest follow-up CT scans in August 2023 confirmed no significant changes at the lesion sites (**Figures 2E, F**). Biochemical testing showed plasma CA19-9 level of 5.37 µ/mL, CEA of 2.29 ng/mL and TBIL of 16.1 μmol/L. The KPS scores remained unchanged.

### Case 2

**Figure 3:**
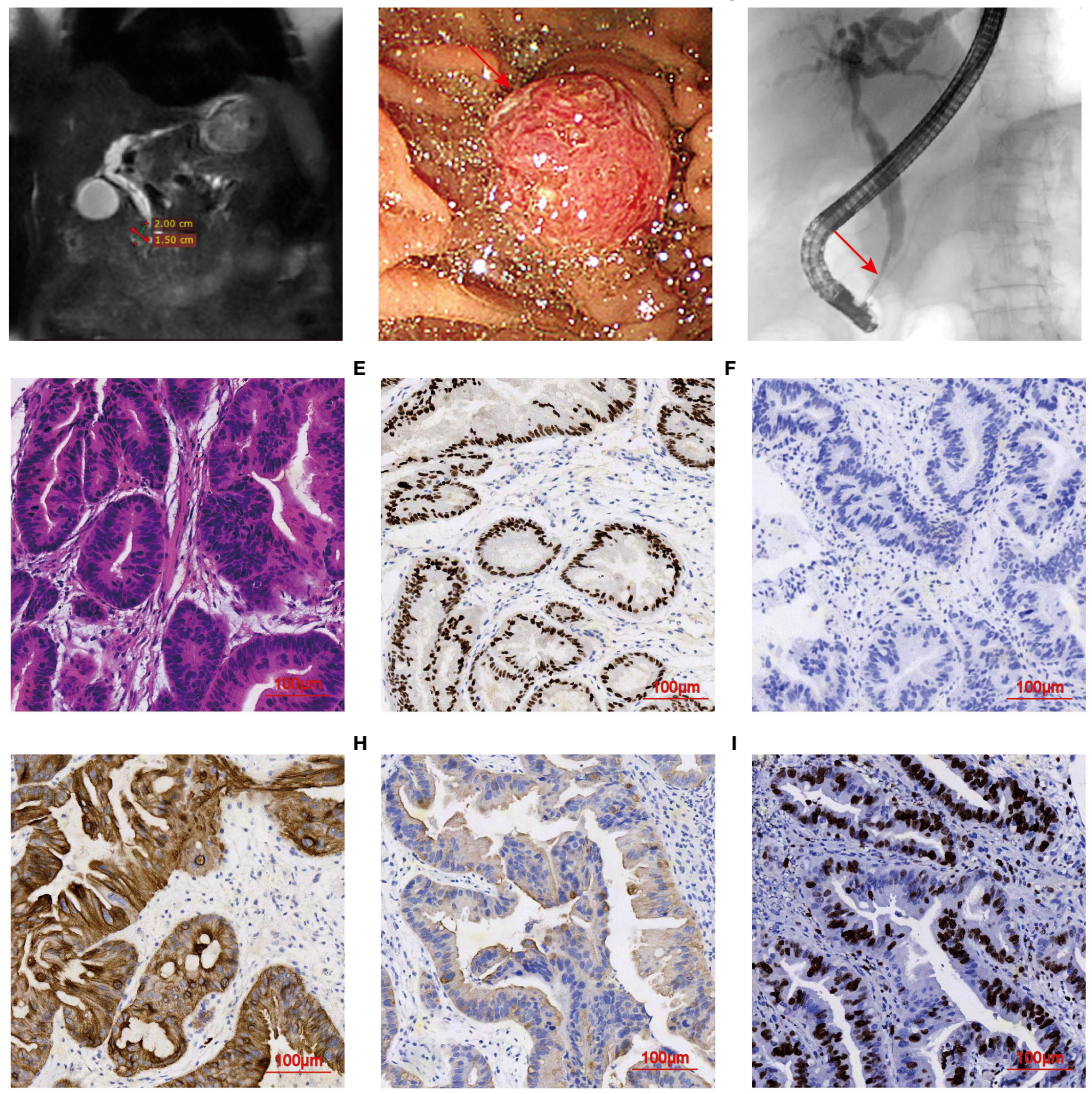
Imaging Features of DPC in case 2. **(A)** MRCP demonstrating a 2.0 cm×1.5 cm mass in the duodenal papilla. **(B, C)** ERCP examination indicating elevated lesion and obstruction of the common bile duct (arrowhead). **(D)** H&E staining of mass tissues (magnification, ×20). **(E–H)** IHC indicating tumors positive for CDX2, CK7, and CK19, and negative for Cerb-B2 (magnification, ×20). **(I)** Apoptosis index. Ki-67 positive rate was about 40%.

In June 2022, an 88-year-old female was admitted to the hospital due to fever and jaundice. History taken from the patient’s son revealed that she had no hypertension, diabetes, hepatitis, prior medical, surgical or psychiatric history. Physical examination revealed vital signs within normal limits. Major abnormal markers of laboratory work-up included the following: white blood cell (WBC) was 19.82×10^9^/L (normal range 3.5-9.5×10^9^/L), neutrophil was 17.6×10^9^/L (normal range 1.8-6.3×10^9^/L), C-reactive protein (CRP) was 18.6 mg/L (normal range < 5 mg/L), alkaline phosphatase level was 527.6 U/L (normal range 50.0-135.0 U/L), gamma-glutamyltranspeptidase was 395.0 U/L (normal range ≤ 38 U/L), TBIL was 88 μmol/L, and CA19-9 was 30.5 U/mL. Laboratory tests confirmed obstructive jaundice and cholangitis. Magnetic resonance cholangiopancreatography (MRCP) identified a 2.0 cm×1.5 cm mass in the duodenal papilla, suggestive of a neoplastic lesion (**Figure 3A**). Furthermore, dilatations of the bile ducts and pancreatic ducts were observed. Subsequent ERCP revealed an obstruction at the terminal of the common bile duct (**Figures 3B, C**). The radiological staging was determined at TxN0M0 by the current 8^th^ edition of the AJCC staging system for Ampulla of Vater cancers. The KPS scores was 50. After the informed consent of the patient and her family, a series of procedures were conducted, including retrograde cholangiopancreatography, duodenal papilla biopsy, and the placement of plastic bile duct stent and pancreatic duct stent, along with a nasobiliary tube. Histopathological examination by hematoxylin and eosin (H&E) staining presented a cribriform architecture and pseudostratification, which was consistent with a diagnosis of moderately differentiated adenocarcinoma of DPC (**Figure 3D**). Immunohistochemical staining showed positivity for CDX2, CK7, and CK19 (focal), with Cerb-B2 negativity (**Figures 3E–H**). A mitotic index was noted with a Ki67-positive rate of 60% (**Figure 3I**). Immunohistochemical markers supported an intestinal subtype of DPC.

**Figure 4:**
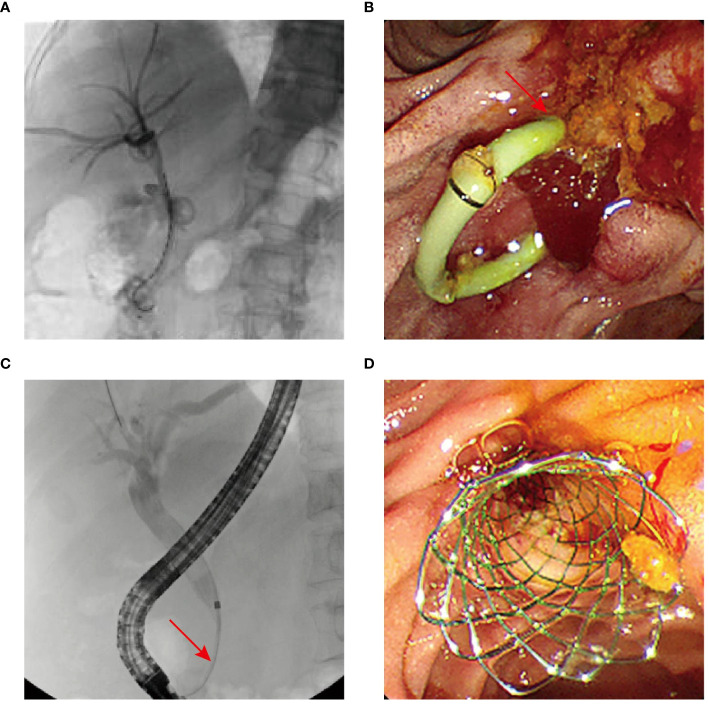
**(A)** Patient in case 2 undergoing PTBD and biliary ^125^I seed implantation. **(B)** Duodenoscope revealing reduction of the lesions. **(C, D)** Biliary stricture (arrowhead) and metal bile duct stent implantation.

**Figure 5:**
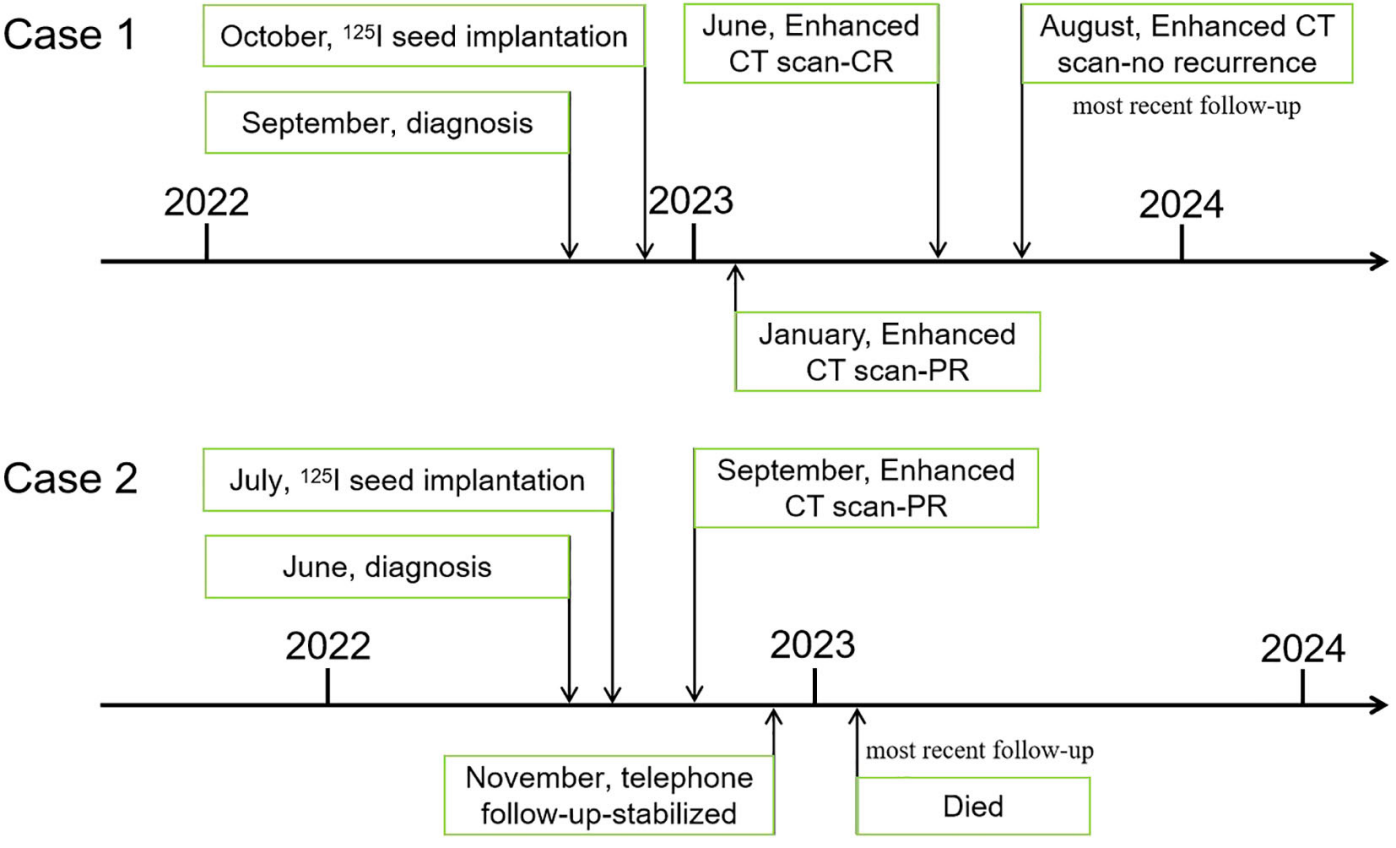
Timelines of major events in both cases.

Following the alleviations of jaundice and fever symptoms, the nasobiliary tube was removed. Percutaneous transhepatic biliary drainage (PTBD) and biliary linear ^125^I seed implantation were performed in July 2022 (**Figure 4A**), alongside anti-infection therapy, nutritional support, and pain management. A significant reduction in the duodenal papilla mass to 1.0 cm × 0.5 cm (with 50% reduction) was observed two months post-treatment (**Figure 4B**). Biochemical blood tests revealed improved levels of alkaline phosphatase (145.1 U/L) and TBIL (36.5 μmol/L), although still mildly elevated. Tumor marker CA19-9 level was normalized. Given the biliary stricture and to avoid the need for a prolonged intubation of drainage tube, procedures were successfully completed, including the extraction of the plastic bile duct, pancreatic duct stents, the biliary drainage tube and biliary linear ^125^I seeds, followed by the placement of a metal bile duct stent (**Figures 4C, D**). After monthly telephone follow-up visits, the patient did not show obvious jaundice and fever symptoms. In November 2022, the patient underwent CT scan in a local hospital, and no tumor progression was observed (patient verbal feedback). Regrettably, the patient succumbed to the Covid-19 in January 2023 (**Figure 5**).

## Discussion

DPC, a subtype of ampullary carcinoma, originates from the ampulla of Vater. The duodenal papilla serves as a crucial anatomical structure in the regulation of bile and pancreatic juice excretion, and it is covered by a multifaceted mucosal layer comprising intestinal, duodenal, and biliary epithelia (11). Given the prognostic and therapeutic implications of histologic and molecular phenotypes, Daphne C Ang has proposed a classification system of ampullary carcinoma based on H&E and IHC findings to distinguish intestinal from pancreaticobiliary subtypes (12). This classification is pivotal, as the pancreaticobiliary type exhibits positive MUC1 staining but lacks positive expressions of CDX2 and MUC2. Conversely, the intestinal subtype is characterized by MUC2 or CK20 positivity and MUC1 negativity. In our study, case 1 was defined as a pancreaticobiliary subtype, whereas case 2 was an intestinal subtype. In addition, further classification was made based on the tumor molecular profile, with *KRAS* and *TP53* mutations being the most common alterations found in DPC. Besides, germline and somatic mutations in *BRCA1/2, ATM*, and other homologous recombination genes were reported in up to 18% of DPCs, potentially predicting an active response to PARP inhibitors and platinum-based therapies (13, 14). In the present case report, however, next-generation sequencing analyses were not performed, as they were beyond the scope of the study.

The treatment of ampullary adenocarcinoma typically involves pancreatoduodenectomy as the primary surgical approach (15). However, more than half cases of ampullary carcinoma are diagnosed till at an advanced stage or an older age, thus precluding the chance of surgical intervention (16). Moreover, standardized recommendations for systemic therapies, predominantly chemotherapy, are scant for unresectable DPC (17). In this context, locoregional therapies may play a role, particularly for locally confined tumors.

Brachytherapy, involving ^125^I seed implantation, acts on various cancers through delivering high radiation doses directly to tumors, while sparing surrounding tissues (18–21). ^125^I seeds emit continuous X-rays and gamma rays to induce apoptosis in tumor cells, with high simplicity, safety, and efficacy (22). Clinical trials have demonstrated promising results of various malignancies treated with intraluminal ^125^I seed implantation (23). In combination with PTBD, intracavitary brachytherapy has been shown to relief clinical symptoms, extend biliary patency and prolong the survival in patients with extrahepatic cholangiocarcinoma, a subtype of ampullary carcinoma (24, 25). In our study, we explored the potential benefits of ^125^I seed implantation in two elderly patients with DPC. In case 1, the treatment effectively controlled local lesions and alleviated intraluminal obstruction, resulting in CR without evidence of recurrence or metastasis. Also, case 2 was well tolerated to ^125^I brachytherapy and achieved PR and >6 month-benefit. Notably, no significant side effects were observed.

**Figure 6:**
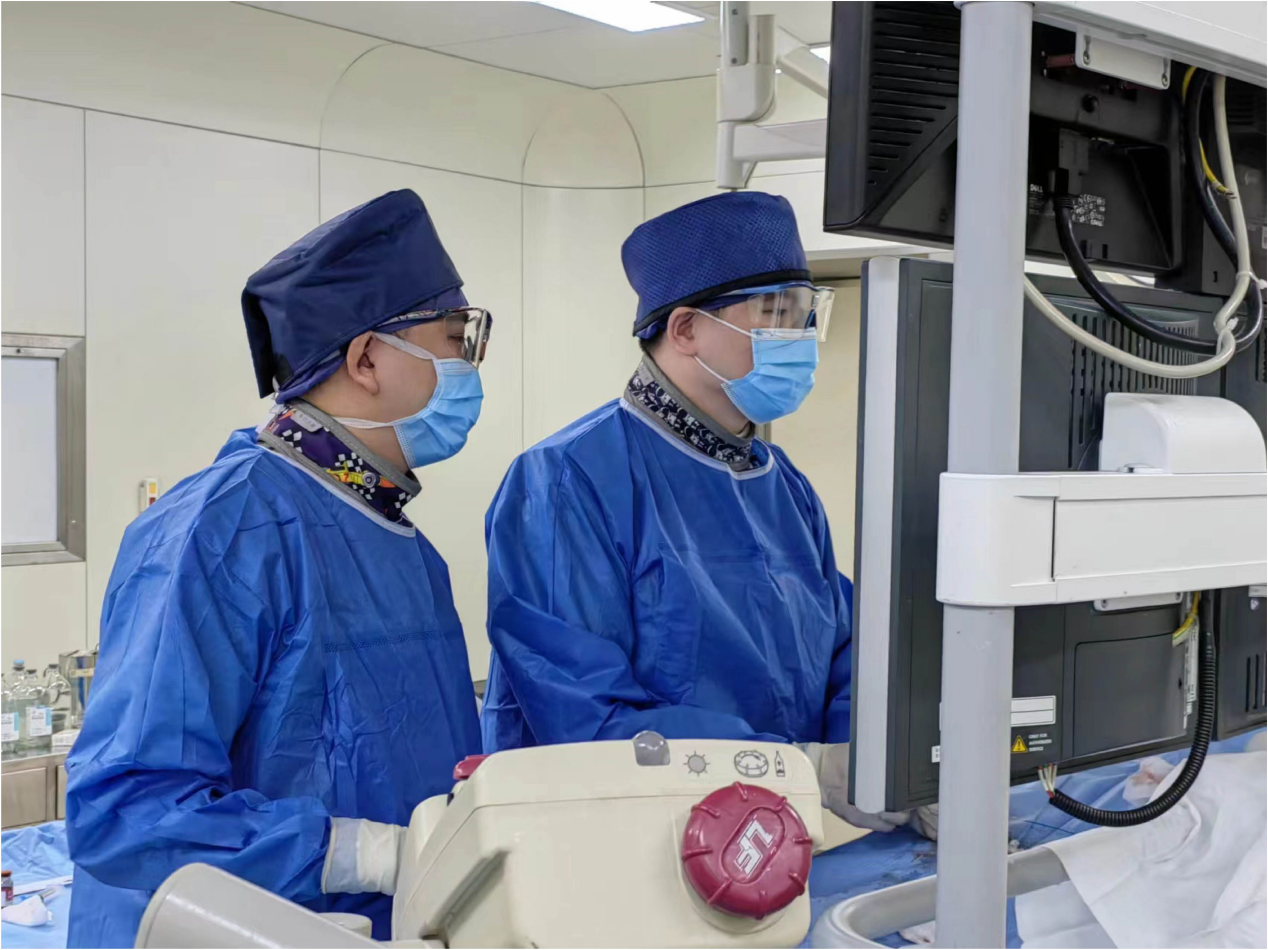
The physician’s protective measures during the implantation of ^125^I seeds in patients include wearing hats, protective glasses, neck gaiter and clothing made of lead.

To address radiation safety issues in ^125^I seed applications, we adhere to a set of standardized methods and procedures strictly. This radiation safety work mainly includes seed receipt, transfer and placement; environmental protection department licensing and regulation, getting approval from the institutional review board and physician radiology credentialing; and the protection of physicians during operation (**Figure 6**), the protection of patients’ families and the setting of seed wards. To prevent radiation exposure to patients and the family members, they were asked to wear lead clothing while in close contact. The half-life of ^125^I seeds in our study was 59.6 days, and patients were asked to avoid an intimate contact with their families for the first time after seed implantation, but they could live together after wearing the protective clothing. The quality of life of patients with implanted seeds can be affected by the disease itself or the increased physical load of heavier lead clothing. Through communication with patients, some feel that wearing protection may receive prejudice from those around them. More analyses are needed in the further.

Our study has several limitations. First, the criteria for ^125^I seed implantation aim to achieve the optimal therapeutic effect while using fewer radioactive sources. However, the distal tumor in our cases was prone to form a low-dose zone, or the exposure of surrounding normal tissue exceeded its maximum tolerated dose, resulting in the less accuracy in radiation dose calculation. Second, as this was a case series without controls, we therefore could not exclude that tumors were clinically indolent. Despite radiological demonstration of brachytherapy activity, the long survival may be attributed to limited tumor aggressiveness. Indeed, although Ki67 levels were remarkably high in this study, tumors were known to be heterogeneous. Estimation of tumor aggressiveness solely by the proliferative index was insufficient. Last, since localized duodenal cancer is extremely rare (most cases are locally advanced or metastatic), further multi-center studies with large sample size and long-term follow up is need to verify the effect of ^125^I seed therapy and/or combined with other therapies (such as chemotherapy, targeted therapy or immunotherapy).

## Conclusion

This is the first report about the favorable outcomes achieved by ^125^I seed implantation in unresectable DPC. Our findings underscore the feasibility and potential activity of ^125^I seed brachytherapy as a treatment modality for DPC.

## Data availability statement

The original contributions presented in the study are included in the article/supplementary material. Further inquiries can be directed to the corresponding author.

## Ethics statement

Written informed consent was obtained from the patients for the publication of this case report. The human images of protective measures also received written informed consent from physicians.

## Author contributions

YZ: Data curation, Writing – original draft. SX: Writing – review & editing. JX: Data curation, Resources, Writing – original draft. SW: Software, Writing – original draft. WY: Data curation, Software, Writing – review & editing. SL: Data curation, Software, Writing – review & editing. GZ: Writing – original draft, Writing – review & editing. TC: Resources, Supervision, Writing – review & editing.
